# Supplementary material for: Aerobic Exercise Alleviates Cardiac Dysfunction Correlated with Lipidomics and Mitochondrial Quality Control
Source: Antioxidants (Basel). 2025 Jun 17;14(6):748. doi: 10.3390/antiox14060748 (PMC12189445; doi:10.3390/antiox14060748)
Supplement: Supplementary file 1 [file antioxidants-14-00748-s001.zip › antioxidants-3589087-supplementary.pdf]

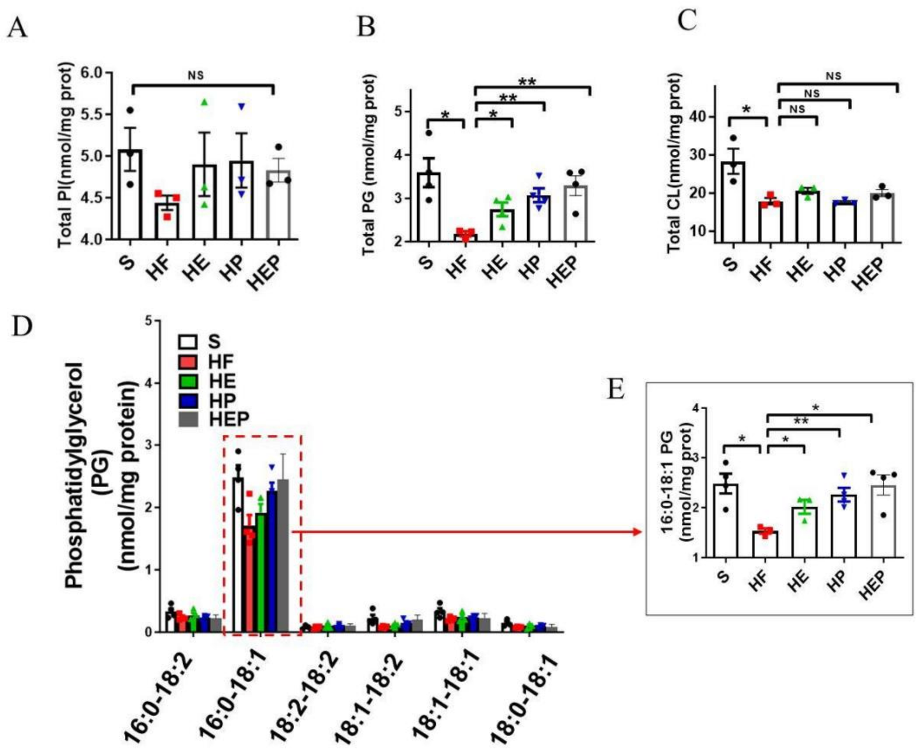

Figure S1 Aerobic exercise restores lipidomic disturbances and cardiolipin acyl composition in right heart cardiomyocytes of heart failure mice. (**A**-**C**) Exercise restored the levels of total PI, PG and CL. (**D,E**) Exercise restored the levels of PG concurrently with increased PG (16:0-18:1). Data are expressed as mean±SD. *, $p < 0.05$; **, $p < 0.01$.
